# A NETWORK ANALYSIS OF FRAILTY USING DATA FROM THE MEXICAN HEALTH AND AGING STUDY

**DOI:** 10.1093/geroni/igz038.2873

**Published:** 2019-11-08

**Authors:** Ricardo Ramírez-Aldana, Carmen García-Peña, Luis Miguel Gutiérrez-Robledo, Lorena Parra-Rodriguez, Juan Carlos Gómez-Verján, Mario Ulises Pérez-Zepeda

**Affiliations:** 1 Instituto Nacional de Geriatria, Mexico City, Mexico; 2 Instituto Nacional de Geriatria, Ciudad de Mexico, Mexico

## Abstract

Frailty remains a challenge in the aging research area with a number of gaps in knowledge still to be filled. New approaches to its study have been proposed, including the one discussed in this article. We tested frailty as through the use of graphical probabilistic models (bayesian networks) with empirical data. Data from the Mexican Health and Aging Study (main data 2012, mortality 2015) was used. Frailty was operationalized with a 35-deficit frailty index (FI). Analyzed nodes were the deficits, plus death and the total score of the FI. The edges, or ties, linking those nodes (set E) were obtained through structural learning, and an undirected discrete graph G (V, E) associated with a discrete graphical probabilistic model (Markov network) was derived. Structural learning was possible through hill-climbing (hc) and PC algorithms. Analyses were performed for the whole population and tertiles of the total FI score. The number of connections within nodes increased according to the tertile level of the total FI score. Groups of interconnected deficits increased as the FI score raised. Almost all deficits related to mobility were interconnected and death was not the most connected node. Frailty behaves as a nonlinear system under the looks of a complex network. Further research should aim to identify the nature of the interactions observed. This could contribute to the development of a conceptual framework that would allow specific interventions to mitigate the consequences of frailty in older adults to be developed.

